# Bullous Pemphigoid Limited to the Hands and Feet: A Rare Case of an Infantile Disease Pattern Seen in an Elderly Patient

**DOI:** 10.7759/cureus.38853

**Published:** 2023-05-10

**Authors:** Aditya Nellore, Nicole Burkemper, Mallory Abate

**Affiliations:** 1 Dermatology, Saint Louis University School of Medicine, Saint Louis, USA; 2 Internal Medicine, St. Luke's Hospital, Chesterfield, USA; 3 Dermatology, The Dermatology Clinic, Baton Rouge, USA

**Keywords:** dermatology, autoimmune, koebnerization, acrally-limited, infantile, bullous pemphigoid

## Abstract

Bullous pemphigoid (BP) is an autoimmune disease that normally presents in older adults, with large bullae distributed over the whole body. Acrally limited BP is a rare pattern of disease that is almost always seen in childhood or infancy. We present an unusual case of a 97-year-old woman with this disease variant and discuss her risk factors which may have contributed to her presentation. Providers should be aware of cases like this so that they can more accurately diagnose and treat their patients.

## Introduction

Bullous pemphigoid (BP) is the most common autoimmune bullous disease of adulthood, presenting with confluent pruritic lesions with large tense bullae. It involves the development of IgG auto-antibodies against hemidesmosomes of the skin’s basement membrane zone, most often the BP180 (BPAg2) and BP230 (BPAg1) proteins [1]. Studies have shown a statistically significant association between neurological disorders, such as dementia and stroke, and the development of BP [2]. BP usually occurs in elderly patients, with a median age of onset of 80 years [2]. Rarely, however, BP presents in childhood or infancy [3]. In such cases, the lesions predominantly occur on the palms and soles rather than the generalized distribution that is typically seen. This type of acrally limited BP is exceedingly rare in adults, with few cases having been reported. Here, we present a case of acrally limited BP in a 97-year-old woman with a history of stroke and dementia.

## Case presentation

**Figure 1:**
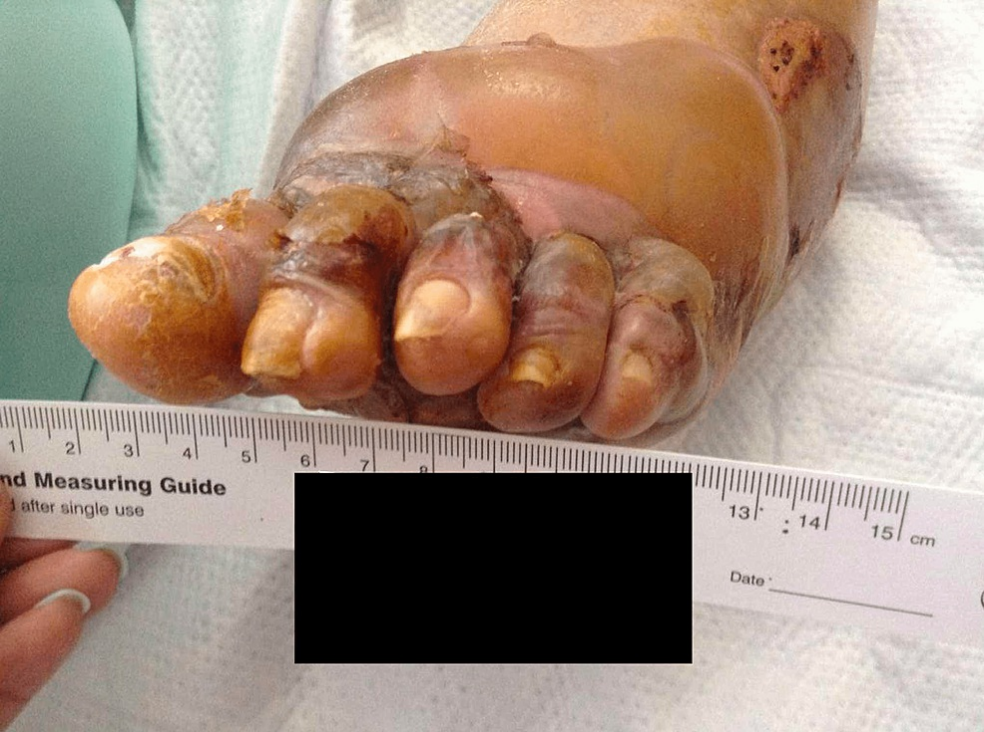
BP of the left foot with tense bullae and denuded erosions

A 97-year-old woman with a history of hypertension, hemiparesis secondary to stroke, and dementia presented with a several-week history of a blistering eruption on her hands and feet. Examination revealed numerous tense bullae of the palms and soles as well as denuded erosions (Figure 1).

The rest of the body was uninvolved. Her current medications were long-standing and included aspirin, famotidine, lisinopril, and vitamin D. Bacterial culture of her lesions was positive for methicillin-sensitive *Staphylococcus aureus.* However, the bullae failed to improve with intravenous antibiotics.

**Figure 2:**
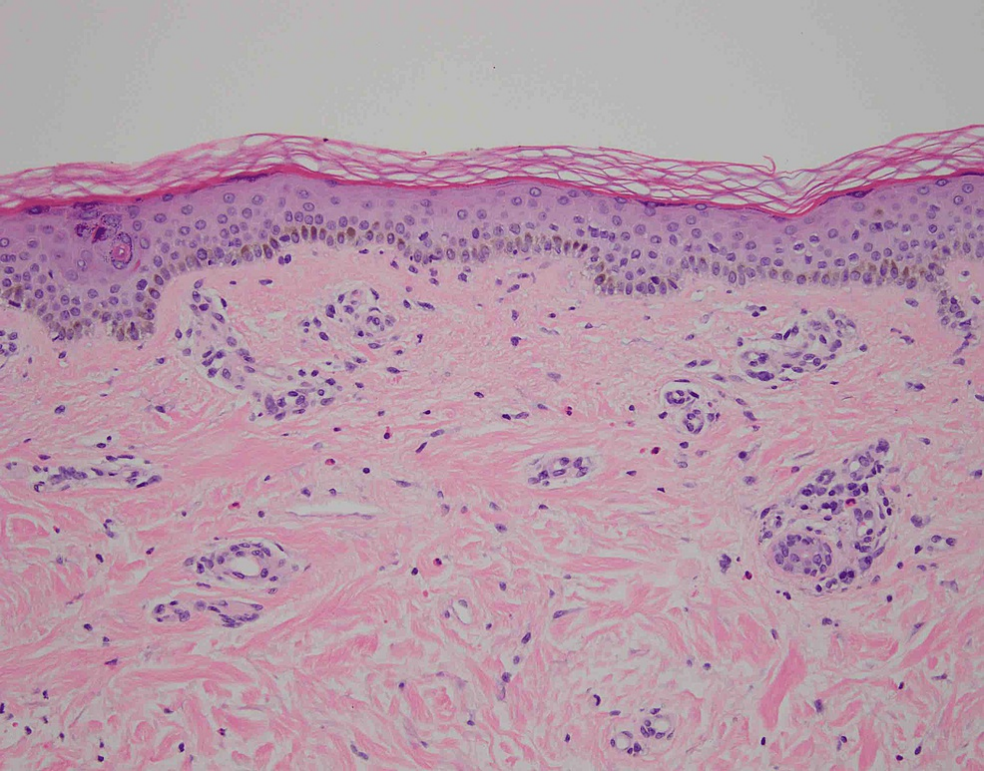
Punch biopsy of lesions on the patient’s left ankle, demonstrating abundant dermal eosinophils. H&E, 20x.

**Figure 3:**
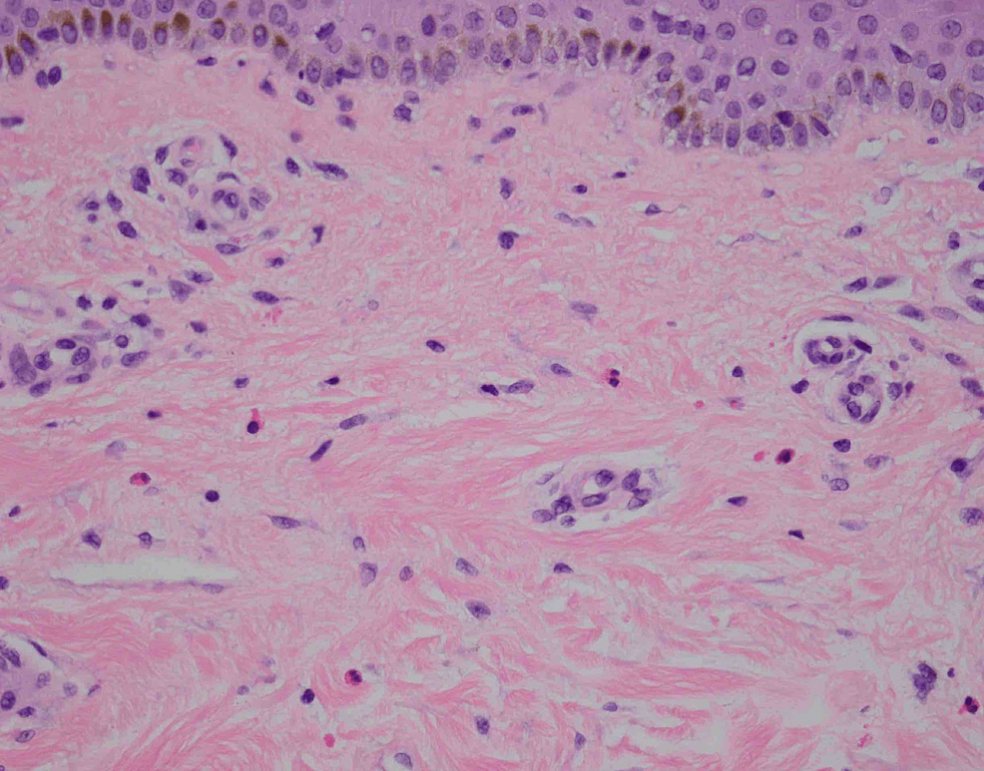
Higher magnification of Figure 2. H&E, 40x.

A punch biopsy of the left ankle was performed for hematoxylin and eosin (H&E) and revealed abundant dermal eosinophils (Figures 2, 3).

Additional biopsy for direct immunofluorescence was negative. Due to the high index of clinical suspicion for autoimmune blistering disease, a serologic testing (ELISA) was conducted and was positive for serum BP 180/230 IgG antibodies, consistent with a diagnosis of BP. The patient significantly improved on topical betamethasone dipropionate ointment and oral prednisone prior to discharge.

## Discussion

Acrally limited or palmoplantar BP is well described in infancy. However, it is rarely described in adults. Although the pathogenesis of generalized BP is well-understood, why this disease sometimes localizes to specific parts of the body is unknown and requires further study. The pathogenesis of localized BP most likely shares some qualities with that of its generalized form, as patients in both groups have auto-antibodies toward the same BP antigens [4]. The difference may lie, then, in local factors, such as BP antigen distribution, tissue trauma, inflammatory injury, or radiation. For example, the acral distribution seen in infants is hypothesized to be due to an age-dependent difference in BP antigen expression in different skin areas [3]. On the other end of the spectrum, certain elderly patients have developed BP on sites of chronic lymphedema or prior radiation therapy [5-7]. Others have reported its occurrence on sites of chronic venous stasis [8]. BP has also been shown to arise on old surgical scars [9-10]. Such cases may be a manifestation of the Koebner phenomenon, which describes the development of skin lesions at sites of tissue damage due to the local physical and physiological changes induced by said damage [11].

On the surface, our patient did not present with any of the aforementioned “local factors,” other than acrally limited sites being more prone to chronic koebnerization However, she did have a history of neurological disease, which has been shown to have a statistically significant association with the development of BP [2]. Several theories to explain this association have been reported. One of the more popular ones involves a neuronal isoform of BP230 (BPAg1) known as dystonin, antibodies that can cross-react with the skin isoform [12]. In fact, this exact process has been shown to occur in a laboratory setting, where significantly more serum samples of patients with BP and neurological diseases recognized dystonin than did samples of patients with BP alone [13]. It is thought that alterations in the central nervous system result in damage to the blood-brain barrier, ultimately leading to exposure of dystonin to the immune system, formation of reactive auto-antibodies, and traversal of those antibodies to the skin [13]. The damage caused by these antibodies could then result in koebnerization, as described previously.

Two neurological diseases that have a high association with the development of BP are dementia and stroke, both of which were found in our patient [2]. Cases of localized BP developing on the hemiplegic or hemiparetic side of a patient’s body following a cerebrovascular accident have been reported [14-15]. This may be due to immune dysfunction of this side of the body, perhaps because of changes in neuropeptide and neurotransmitter levels [16]. Our patient developed paresis on both sides of the body following her stroke, which could be a contributing factor to her development of BP on all four limbs. Other cases have been reported of patients suffering from dementia developing some form of BP [2,15]. It has been shown that dementia patients have abnormally high levels of anti-BP-180 and anti-BP-230 IgG antibodies and demonstrate a BP pattern on indirect immunofluorescence [17]. These findings support the idea that patients with dementia are, indeed, at a higher risk of BP.

## Conclusions

Because our patient had a history of both aforementioned predisposing conditions, it may not be surprising that she eventually developed BP. However, further study is necessary to explain her unusual palmoplantar distribution, as is more typically isolated to cases of BP seen in infancy. Infants with this disease often have a much better response to treatment than adults, with lower rates of relapse. Thus, having acrally limited lesions may have important treatment and prognostic implications, warranting a better understanding of the pathogenetic similarities between infantile BP and cases such as ours.
